# Rab27A Is Present in Mouse Pancreatic Acinar Cells and Is Required for Digestive Enzyme Secretion

**DOI:** 10.1371/journal.pone.0125596

**Published:** 2015-05-07

**Authors:** Yanan Hou, Stephen A. Ernst, Edward L. Stuenkel, Stephen I. Lentz, John A. Williams

**Affiliations:** 1 Department of Molecular and Integrative Physiology, University of Michigan, Ann Arbor, Michigan, United States of America; 2 Department of Cell and Developmental Biology, University of Michigan, Ann Arbor, Michigan, United States of America; 3 Internal Medicine, University of Michigan, Ann Arbor, Michigan, United States of America; Centro Nacional de Investigaciones Oncológicas (CNIO), SPAIN

## Abstract

The small G-protein Rab27A has been shown to regulate the intracellular trafficking of secretory granules in various cell types. However, the presence, subcellular localization and functional impact of Rab27A on digestive enzyme secretion by mouse pancreatic acinar cells are poorly understood. *Ashen* mice, which lack the expression of Rab27A due to a spontaneous mutation, were used to investigate the function of Rab27A in pancreatic acinar cells. Isolated pancreatic acini were prepared from wild-type or *ashen* mouse pancreas by collagenase digestion, and CCK- or carbachol-induced amylase secretion was measured. Secretion occurring through the major-regulated secretory pathway, which is characterized by zymogen granules secretion, was visualized by Dextran-Texas Red labeling of exocytotic granules. The minor-regulated secretory pathway, which operates through the endosomal/lysosomal pathway, was characterized by luminal cell surface labeling of lysosomal associated membrane protein 1 (LAMP1). Compared to wild-type, expression of Rab27B was slightly increased in *ashen* mouse acini, while Rab3D and digestive enzymes (amylase, lipase, chymotrypsin and elastase) were not affected. Localization of Rab27B, Rab3D and amylase by immunofluorescence was similar in both wild-type and *ashen* acinar cells. The GTP-bound states of Rab27B and Rab3D in wild-type and *ashen* mouse acini also remained similar in amount. In contrast, acini from *ashen* mice showed decreased amylase release induced by CCK- or carbachol. Rab27A deficiency reduced the apical cell surface labeling of LAMP1, but did not affect that of Dextran-Texas Red incorporation into the fusion pockets at luminal surface. These results show that Rab27A is present in mouse pancreatic acinar cells and mainly regulates secretion through the minor-regulated pathway.

## Introduction

The small G protein Rab27A has been demonstrated to play important roles in mediating intracellular organelle movement and secretion in various cell types. Mutations of Rab27A are causal to type 2 Griscelli Syndrome, a rare, autosomal recessive disorder that results in pigmentary dilution of the skin and hair with the presence of large clumps of pigment in hair shafts and an accumulation of melanosomes in melanocytes [1]. A single point mutation in the mouse orthologue of Rab27A is responsible for the phenotypes in *ashen* mice, including uneven release of pigment into the hair bulb and a lightened coat color [2]. It has been shown that mutation of Rab27A in melanocytes blocks normal actin-based migration of melanosomes to the cell periphery [3–6]. Rab27A has also been shown to regulate the exocytosis of secretory granules in adrenal chromaffin cells. Rab27A and its effector MyRIP (also known as Slac2-c) were reported to be associated with large dense core granules in adrenal chromaffin and pheochromocytoma PC12 cells and to control the secretory activity in a manner that depends on the state of the actin cortex [7]. Overexpression of Rab27A in PC12 cells promoted high KCl-dependent secretion of neuropeptide Y [8]. Rab27A was also found to play a key role in the docking step of dense-core vesicle exocytosis in PC12 cells; silencing of Rab27A significantly decreased the number of dense-core vesicles docked to the plasma membrane without altering the kinetics of individual exocytotic events [9]. In pancreatic beta-cells, Rab27A was shown to mediate the tight docking of insulin granules to the plasma membrane upon high glucose stimulation. *Ashen* mice showed glucose intolerance without signs of insulin resistance in peripheral tissues or insulin deficiency in the pancreas. The docking of insulin granules on the plasma membrane and the replenishment of docked granules during glucose stimulation were markedly reduced in *ashen* mouse pancreatic islets [10]. A recent study showed that GTP/GDP nucleotide cycling of Rab27A is essential for generation of the functionally defined immediately releasable pool (IRP) and central to regulating the size of the readily releasable pool (RRP) of insulin-containing secretory granules in pancreatic beta-cells [11].

The other isoform of Rab27, Rab27B, has been found to mediate exocytosis in a large variety of secretory cells. We have previously reported Rab27B was abundantly expressed on the zymogen granule (ZG) membrane of rat pancreatic acinar cells [12,13]. Over-expression of constitutively active Rab27B enhanced CCK- induced amylase release from isolated rat pancreatic acini, while dominant negative Rab27B inhibited amylase release [14]. These results demonstrate that Rab27B is present on ZGs and plays an important role in regulating acinar exocytosis through ZG secretory pathway, also known as the major regulated secretory pathway [12–14]. In addition to the major regulated pathway, two other secretory pathways, the minor-regulated pathway (MRP) and constitutive-like pathway (CLP), have been identified in pancreatic and parotid acinar cells [15–18]. In parotid acinar cells, together with ZG pathway, CLP and MRP regulate exocytosis at different levels, with CLP being active continually and the MRP responding to lower level stimulation [15]. CLP and MRP are responsible for a small fraction of secretory protein release, compared with the ZG pathway. Both CLP and MRP originate from immature granules; after transition at an endosome-like compartment, vesicles of both pathways are then transported to apical membrane [15,17]. However, the proteins involved in mediating the final step of this process are largely unknown.

In the current study, we show that Rab27A is present in mouse pancreatic acinar cells and partially co-localizes with Rab27B to the zymogen granule membrane. Rab27A deficiency did not significantly affect the morphology of acini, but showed decreased amylase release upon stimulation by secretagogues. Fluorescent tracking of secretion showed that Rab27A knockout did not affect the zymogen granule secretory pathway, but rather the lysosomal-like secretory pathway. These findings indicated that Rab27A is present in pancreatic acinar cells and plays a different role than Rab27B in regulating secretion in pancreatic acinar cells.

## Materials and Methods

The plasmids encoding GST-SHD (Synaptotagmin-like protein (Slp)-homology domain) and GST-Rim (Rab3-interacting molecule)) (amino acids 1–399) fusion proteins were provided by Dr. Hisanori Horiuchi [19] and Dr. Ronald W. Holz (University of Michigan), respectively. Glutathione-Sepharose 4B beads were purchased from GE-Healthcare (Piscataway, NJ). Antibodies that were used are as follows: rabbit polyclonal antibodies against Rab27B (Cat. No. 168 103, 1:4,000 dilution) and Rab27A (Cat. No. 168 013, 1:2,000 dilution) from Synaptic Systems (Goettingen, Germany); rabbit polyclonal antibodies against LAMP1 (Cat. No. ab24170, 1:1,000 dilution) and VDAC (Cat. No. ab15895, 1:1,000 dilution) from Abcam (Cambridge, MA); anti-Bip monoclonal antibody from BD Biosciences (San Jose, CA, Cat. No.610978, 1:2,000 dilution); rabbit polyclonal anti-Calnexin antibody from Stressgen Bioreagents (Ann Arbor, MI, Cat. No. ADI-SPA-860, 1:2,000 dilution); rabbit anti-Rab3D antisera (1:5,000 dilution for WB, 1:500 for IF) was a gift from Dr. Mark McNiven (Mayo Clinic, Rochester, MN); rabbit polyclonal antibodies against Rab 6 (Cat. No. sc-130, 1:200 dilution) and Rab11 (Cat. No. sc-9020, 1:200 dilution) from Santa Cruz (Dallas, TX); rabbit polyclonal antibodies against elastase (Cat. No. 100–468, 1:2,000 dilution) and ribonuclease (Cat. No. 100–4188, 1:5,000 dilution) were purchased from Rockland Immunochemicals (Gilbertsville, PA); rabbit polyclonal anti-chymotrypsin (Cat. No. 20C-CR1095R, 1:2,000 dilution) and mouse monoclonal anti-lipase (Cat. No. 10-L50A, 1:1,000 dilution) antibodies from Fitzgerald Industries International (Acton, MA); mouse monoclonal anti-GFP antibody (Cat. No. sc-9996, 1:50 dilution for IF) from Santa Cruz Biotechnology (Dallas, TX); rabbit polyclonal anti-amylase antibody from Sigma (Cat. No. A-8273, 1:10,000 dilution for WB, 1:200 for IF); goat polyclonal anti-carboxypeptidase A antibody (Cat. No. AF2765, 1:1,000 dilution) from R&D Systems (Minneapolis, MN). Collagenase NB8 was purchased from SERVA (Heidelberg, Germany), 8-pCPT-2’-O-Me-cAMP from BioLog (Bremen, Germany), Lysine Fixable Dextran-Texas Red 3000 MW from Molecular Probes (Eugene, OR). All other reagents were from Sigma. Adenovirus encoding cyan fluorescence protein (CFP)-tagged Rab27A was constructed by sub-cloning CFP-Rab27A into adenoviral shuttle vector pAdTrack-CMV, and then recombined with adenoviral backbone vector pAdEasy-1, as previously described [20].

### Ethics Statement

All the experimental protocols were approved by the University of Michigan Committee on the Use and Care of Animals (UCUCA). Total number of animals used in this study is 110–130, including ICR, C3H/HeSnJ and *ashen* mice. Carbon dioxide inhalation was used for euthanasia.

### Isolation, adenoviral infection of pancreatic acini and analysis of amylase secretion

Pancreatic acini were isolated from 5–7 week old male ICR mice, or 8–12 week old male wild-type C3H/HeSnJ and *ashen* mice by collagenase digestion [21]. When appropriate, adenoviral infections were performed on isolated acini as previously described [20,22]. Freshly made acini were used for amylase release after pre-incubation at 37°C for 30 min; overnight cultured acini were harvested in Hepes-Ringer buffer and then incubated with secretagogues. After incubation with different secretagogues at various concentrations for 30 min, the acinar suspension was centrifuged for 20 s in a microcentrifuge and the supernatant was assayed for amylase activity using Phadebas reagent (Amersham Biosciences and Upjohn) as previously described [21,23]. The pellets were collected and lysed for DNA content measurement using Qubit 2.0 fluorometer and Qubit dsDNA HS Assay Kit (Life Technologies, Eugene, OR). Secretion was either expressed as a percentage of initial acinar amylase total content or units per milligram DNA.

### Assessment of Pancreatic Morphology and Ultrastructure

Hematoxylin and eosin staining of paraffin sections was performed following standard protocol. Electron microscopy was performed as described previously [24]. Briefly, pancreas was minced with razor blade and fixed for 2 hours with a mixture of 2% glutaraldehyde and 2% formaldehyde in phosphate-buffered saline (PBS), post-fixed for 45 minutes with 1% OsO_4_, and then dehydrated and embedded in Epon. Ultrathin sections were stained with uranyl acetate and lead citrate; images were recorded digitally using a Philips CM-100 electron microscope.

### Tissue Fractionation

Zymogen granules, zymogen granule membranes, cytosol and microsomes were purified from ICR mouse pancreas as previously described [25]. The mouse pancreas was homogenized in buffer containing 0.25 M sucrose, 25 mM MES (2-Morpholinoethanesulfoni acid, monohydrate) pH 6.0, 0.1 mM MgSO_4_, 2 mM EGTA and 0.1 mM phenylmethylsulfonyl fluoride (PMSF). Unbroken cells and nuclei were removed by centrifugation at 200 g for 10 min at 4°C. Postnuclear supernatant was centrifuged at 2,000 g for 10 min to obtain a pellet enriched in ZGs and mitochondria. ZGs were further purified by Percoll gradient centrifugation. The 2,000 g supernatant was centrifuged at 10,000 g for 10 min to produce a pellet enriched in mitochondria; the supernatant was further spun at 100,000 g for 1 hr to separate microsomal and cytosolic fractions. The ZGs were washed with homogenization buffer and resuspended in ZG lysis buffer containing 150 mM sodium acetate, 10 mM MOPS, pH 7.0, 27 μg/ml Nigericin, 0.1 mM MgSO_4_, 0.1 mM PMSF, supplemented with protease inhibitors cocktail, and incubated at 37°C for 15 min. The lysate was centrifuged at 38,000 rpm (100,000 g) for one hour in a Beckman ultracentrifuge using a Ti 70.1 rotor to pellet the ZG membrane. The supernatant was saved as ZG content. The ZG membrane pellet was sequentially washed with 250 mM KBr and 0.1 M Na_2_CO_3_ (pH 11.0). Equal amount of totally protein from different fractions were separated on SDS-PAGE and blotted with indicated antibodies.

### Apical Exocytosis Labeling with Dextran-Texas Red

To quantify the secretion that occurs through the zymogen granule secretory pathway, we adopted the technique pioneered by Nemoto et al. [26] and modified by Turvey and Thorn [27], to visualize exocytotic membrane fusions with a luminal tracer. We incubated 500 μL of a suspension of mouse acini freshly isolated from WT or *ashen* mice at 37°C in HEPES-buffered Ringer’s solution containing 1 mg/mL Texas Red labeled dextran (3000 Da), which had been modified with fixable lysine residues. After 5 min a small aliquot of carbachol (CCh) was added to the solution to yield a final concentration of 3 μM. After another 5 min incubation at 37°C, the acini were collected and fixed by rinsing twice with fresh 4% formaldehyde in PBS followed by incubation at room temperature for 30 min. After fixation, the acini were rinsed once with PBS and incubated with Alexa Fluor 488-conjugated Phalloidin for 20 min, to label filamentous actin which appears in acinar cells primarily as a sub-luminal membrane band and is known to associate with granules during exocytosis [28]. The acini were then rinsed once, resuspended in PBS, and transferred to 35 mm tissue culture dish with cover glass bottom (World Precision Instruments, Inc.) for imaging with Olympus FluoView FV500 confocal microscope. Images were deconvolved using AutoQuant X2 and then the dextran-Texas Red filled omega structures were quantified with Imaris software (Bitplane, Zurich, Switzerland). The numbers of dextran labeled omega figures coated with phalloidin along the apical lumen were counted and each sphere was deemed as one exocytosis event. The length of the apical lumen visualized by sub-luminal phalloidin staining was measured using Imaris in a 3D manner. The total number of exocytosis events in each image was normalized to the corresponding apical lumen length.

### Apical Lumen Surface Labeling of LAMP1

The apical lumen surface labeling of LAMP1, as a measure of endosomal/lysosomal secretion, was performed as previously described [17]. Fresh acini from WT or *ashen* mice were incubated with 30 pM CCK at 37°C for 5 min. Cells were gently pelleted on ice and resuspended with ice-cold fresh media containing mouse anti-LAMP1antibody (1:20, Enzo Life Sciences, Cat. #ADI-VAM-EN001), which reacts with the luminal epitope of LAMP1, and gently rotated on a wheel for 2 hr at 4°C. Cells were then fixed using 2% formaldehyde in PBS at room temp for 10 min, rinsed with PBS, pelleted and resuspended in blocking buffer devoid of Triton X-100 (PBS, 3% bovine serum albumin, 2% goat serum, 0.7% cold-water fish skin gelatin), which contained Alexa Fluor 594-conjugated anti-mouse IgG (1:100) for 1 hr and Alexa Fluor 488-conjugated Phalloidin at room temp for 20 min. Cells were rinsed once, resuspended in PBS, and transferred to 35 mm tissue culture dish with cover glass bottom for imaging with the inverted Olympus FluoView FV500 confocal microscope. Z-stack images were taken with a thickness of 0.5 μm per optical section. Images were then processed with Imaris software (Bitplane, Zurich, Switzerland). Apical lumen area was cropped based on phalloidin staining. The intensity of LAMP1 immunoreactivity encapsulated within the phalloidin was quantified and normalized to the acinar volume obtained from the z-stack. Representative images were processed by Image J.

### Statistical Analysis

Statistical significance was determined by the Student’s *t*-test.

## Results

### Expression of Rab27B is increased in *ashen* mouse acini

**Fig 1:**
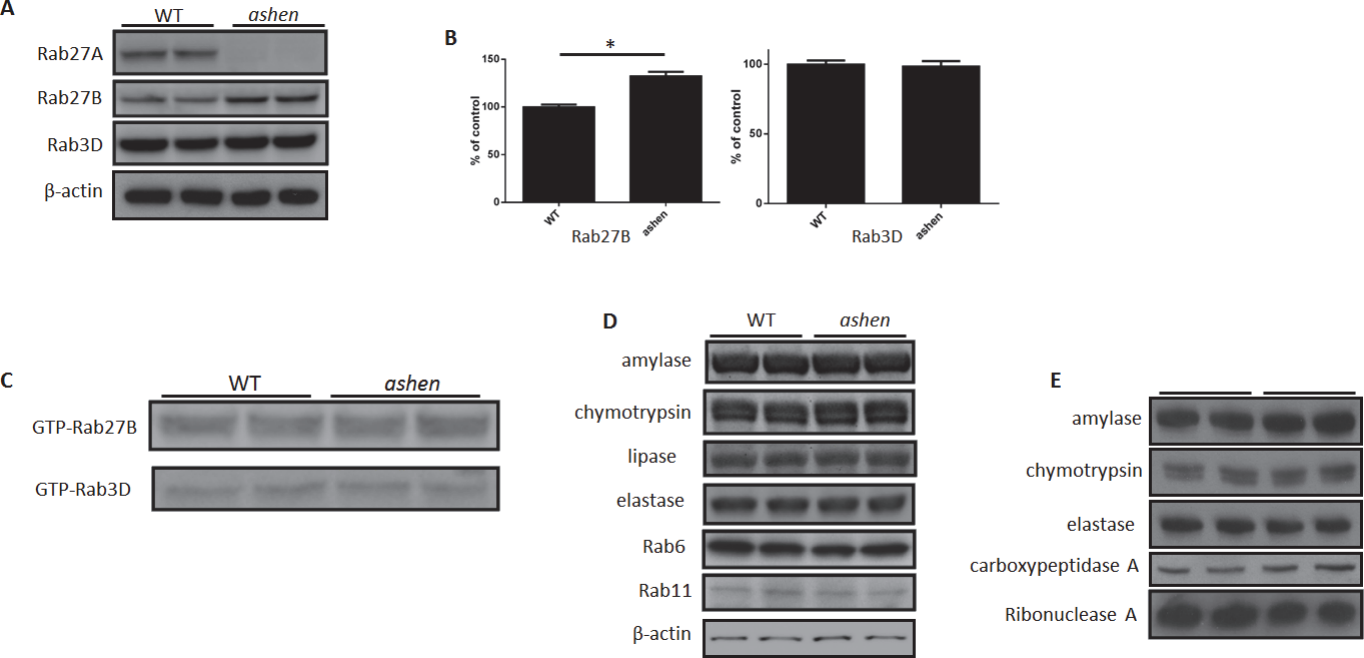
Rab27A deficiency caused increased expression of Rab27B, but did not affect its activity. (**A** and **B**) Examples of total lysates of isolated pancreatic acinar cells from wild-type C3H/HeSnJ or *ashen* mice were analyzed by western blot. Each lane represents samples from one mouse. (**B**) Densitometry analysis on the western blot results from all samples run as in (A). The results are mean ± SE from five mice of each genotype. *P < 0.05. (**C**) Active form of Rab27B and Rab3D at basal level in isolated acini was examined by GST-SHD and GST-Rim pulldown, respectively. Pulldown fractions were analyzed by western blot. This experiment was repeated three times with similar results. (**D**) The expression of major digestive enzymes (amylase, chymotrypsin, lipase, and elastase) and other Rab proteins (Rab6 and Rab11) was also not changed in western blots on lysates from isolated *ashen* mouse acinar cells. (**E**) The expression of major digestive enzymes (amylase, chymotrypsin, elastase, carboxypeptidase A and ribonuclease A) was also not changed in western blots of purified *ashen* mouse zymogen granules.

To examine the effect of the absence of Rab27A on the expression of other proteins in pancreatic acinar cells, we isolated the acini from wild-type C3H/HeSnJ mice (WT) and *ashen* mice. While Rab27A was confirmed as knocked out in *ashen* mouse acini, the expression of Rab27B was increased by 32.5% ± 4.4% and that of Rab3D was unchanged, compared with WT values (Fig 1A and 1B). In spite of a higher expression of the Rab27B in *ashen* acini, the GTP-bound active forms of Rab27B and Rab3D were unchanged from control (Fig 1C). The expression of major digestive enzymes (amylase, chymotrypsin, lipase and elastase) and other Rab proteins, e.g. Rab6 and Rab11, was also not changed in the *ashen* acini (Fig 1D). The content of major digestive enzymes in *ashen* mouse zymogen granules was also not changed compared to WT (Fig 1E).

### Rab27A deficiency did not affect the morphology and ultrastructure of pancreatic acinar cells

**Fig 2:**
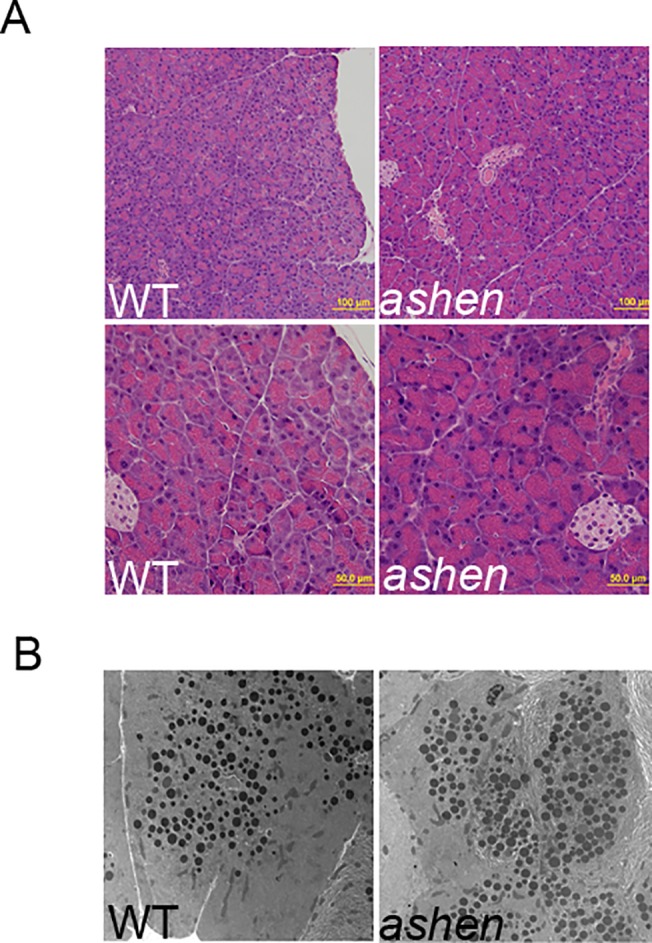
Rab27A deficiency did not affect the morphology of pancreas. (**A**) Pancreas tissues freshly obtained from WT or *ashen* mice were fixed, embedded with paraffin, sectioned and stained with hematoxylin and eosin. Images were taken using a 20X (upper panel) and 40X (lower panel) objective lens. (**B**) Pancreas tissues from WT and *ashen* mice were processed for electron microscopy as described in Material and Methods. Images were obtained at the magnification of 3400X.

To compare the morphology of pancreas from WT and *ashen* mice, paraffin-embedded pancreas tissues were sectioned and stained with hematoxylin and eosin. No obvious difference in overall acinar cell morphology was observed between WT and *ashen* mice (Fig 2A). Ultrastructure of WT and *ashen* pancreas was also assessed by electron microscopy. *Ashen* acinar cells showed normal ultrastructure; no obvious abnormalities in size, number and localization of electron dense secretory granules were detected in *ashen* acinar cells, compared with WT (Fig 2B).

### Rab27A deficiency did not affect the localization of Rab27B and Rab3D

**Fig 3:**
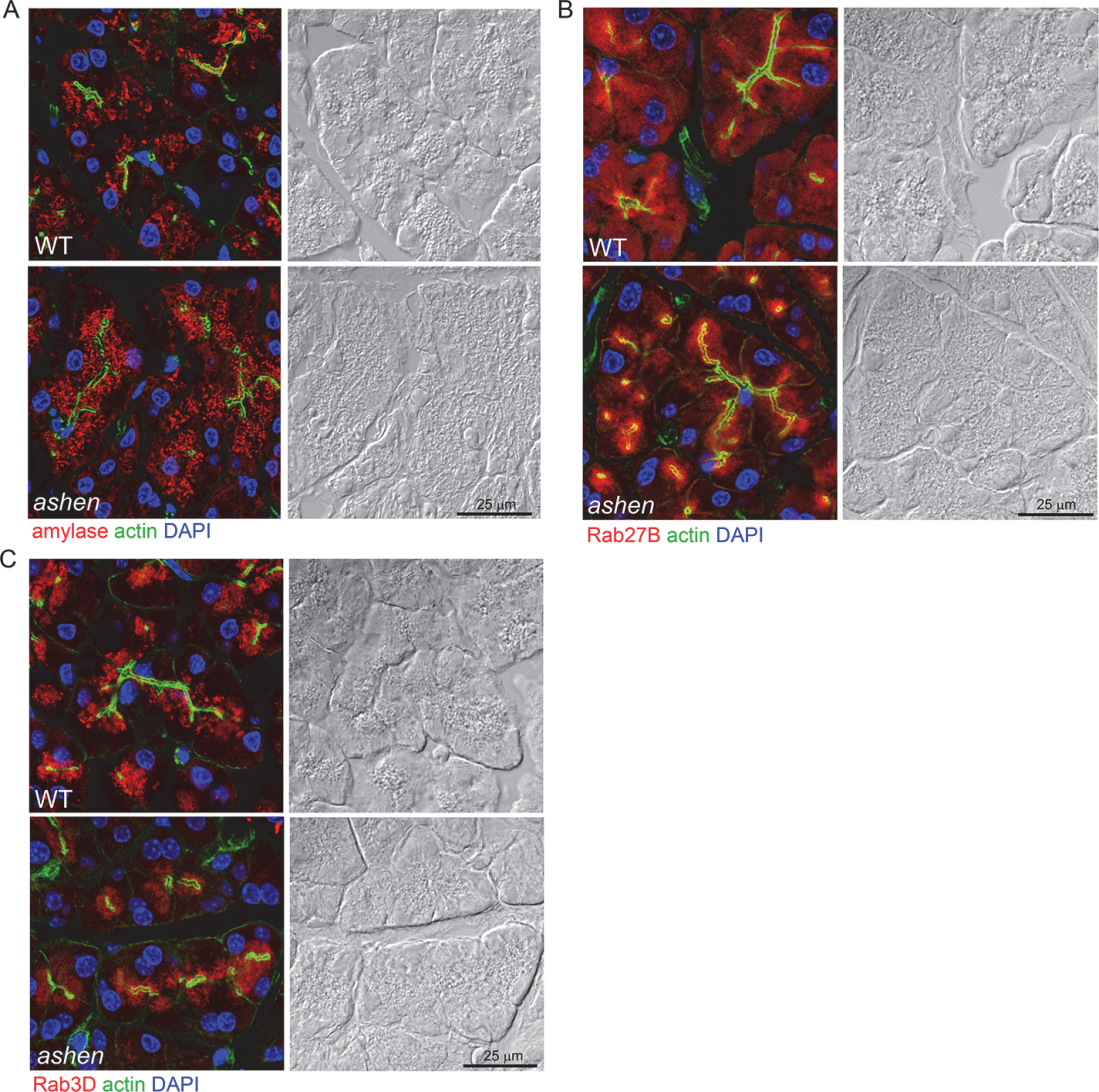
Rab27A deficiency did not affect the localization of zymogen granules, Rab27B and Rab3D. Pancreas tissues were cryosectioned at 10 μm, stained with phalloidin to label actin (green), DAPI to label nuclei (blue) and anti-amylase (A), anti-Rab27B (B) or anti-Rab3D (C) antibodies (red), respectively. Images were obtained using confocal microscopy. Each panel is paired with the Nomarski image for the same section. Scale bar = 25 μm.

To test whether the knockout of Rab27A affects the localization of zymogen granules, Rab27B and Rab3D, immunofluorescence localization of these proteins in cryostat sections was examined in pancreas tissues from WT and *ashe*n mice. The secretory granules positive for amylase, Rab27B or Rab3D showed polarized localization in the apical compartment of acini in both WT and *ashen* pancreas. No obvious difference in the fluorescence intensity of amylase, Rab27B or Rab3D was observed between WT and *ashen* mice (Fig 3A–3C).

### Rab27A showed partial co-localization with zymogen granules

**Fig 4:**
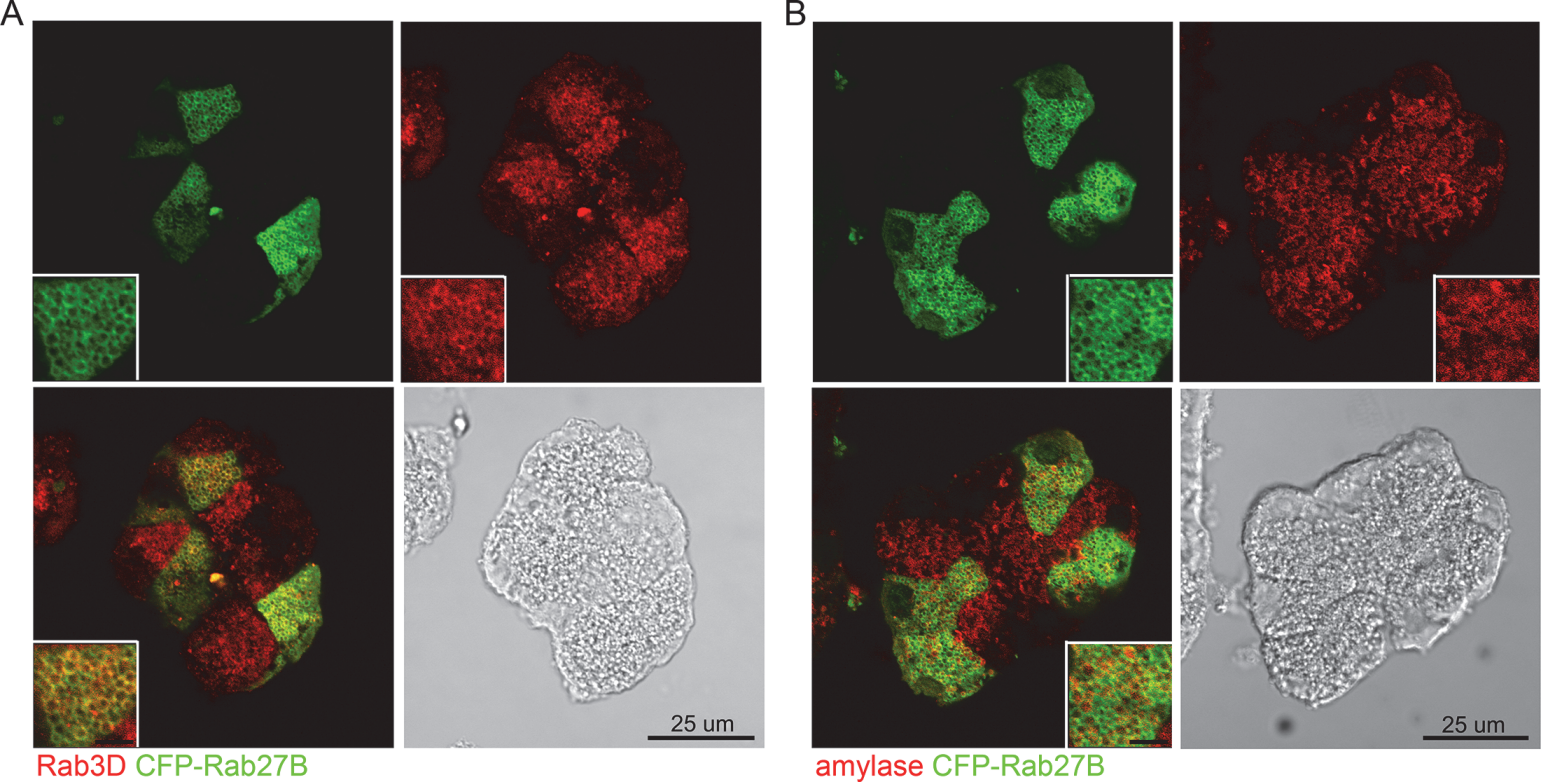
Rab27A showed partial co-localization with zymogen granules. Expression of CFP-Rab27A was mediated by adenoviral infection at a titer of 5×10^6^ pfu/mL to avoid excessive expression of exogenous protein. Therefore the acinar cells were partially positive for CFP-Rab27A. Anti-GFP antibody was used to enhance the CFP-Rab27A signal and doubled stained with anti-Rab3D (**A**) or anti-amylase antibody (**B**), respectively. Fluorescence images were obtained by confocal microscopy. Scale bars in the insets represent 5 μm.

The localization of Rab27A was examined by overexpressing a cyan fluorescence protein (CFP)-tagged Rab27A in isolated mouse acini via adenoviral infection, because available antibodies to Rab27A did not work for immunofluorescence. Double staining was performed using anti-GFP antibody (to enhance the CFP signal) together with anti-Rab3D or anti-amylase antibodies on fixed sections prepared from overnight cultured acini. In the acinar cells positive for CFP-Rab27A, Rab27A fluorescence showed partial co-localization with Rab3D and amylase (Fig 4A and 4B). These observations indicated that Rab27A is present on the zymogen granules, but in addition it was more broadly distributed than Rab3D, which showed specific localization to the zymogen granules.

### Rab27A showed different subcellular localization than Rab27B and Rab3D

**Fig 5:**
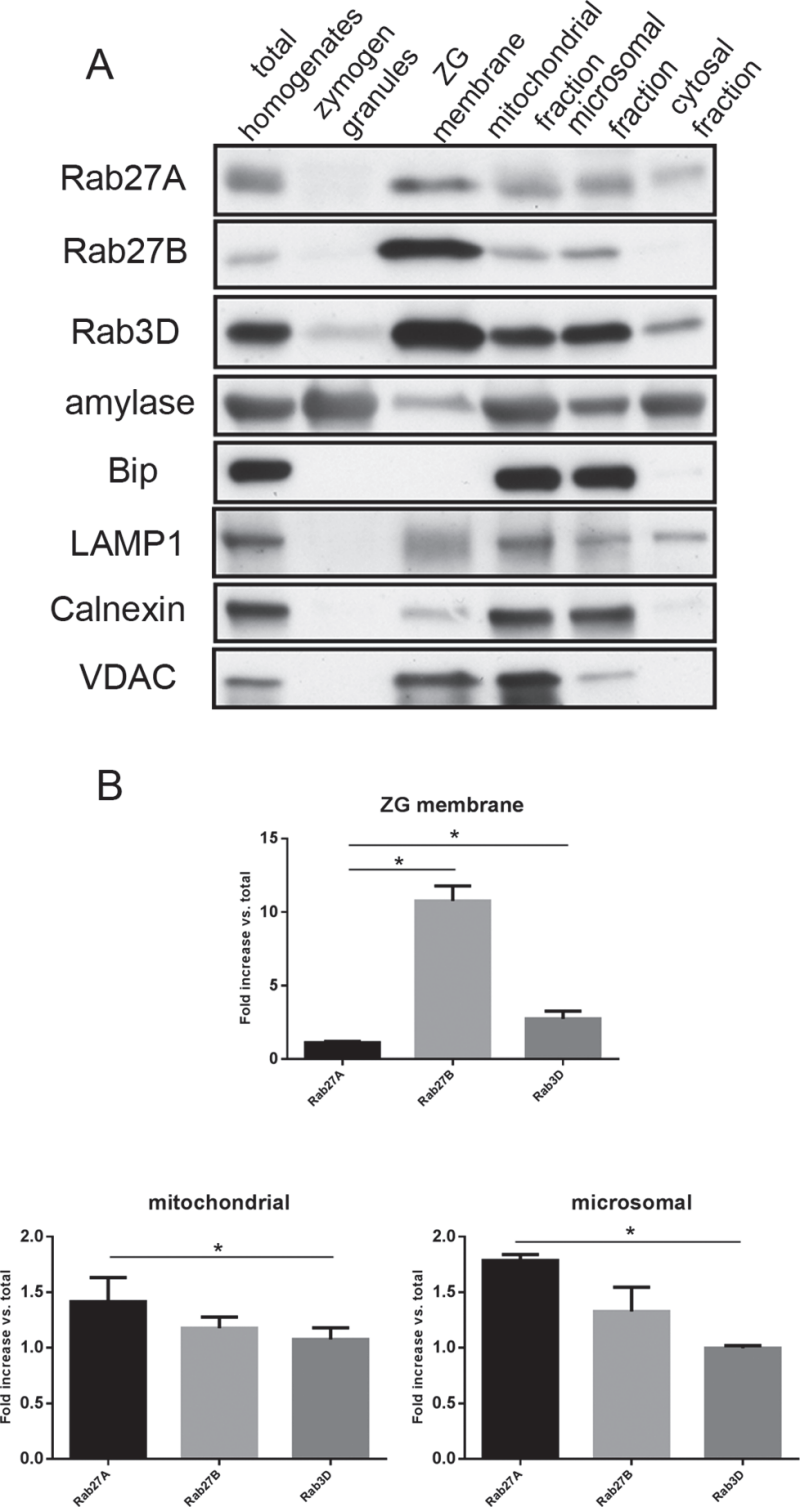
Rab27A exhibited different subcellular localization than Rab27B and Rab3D. (**A**) Cell fractionation samples from ICR mouse pancreas were loaded at 10 μg/lane and separated by PAGE. Antibodies against small G-proteins and marker proteins of different subcellular compartments were used in western blots. (**B**) Densitometry analysis on the western blot results from purified ZG membrane, mitochondrial and microsomal fractions shown in (A). The results are mean ± SE from three separate cell fractionation experiments. Pancreas tissues from 5–6 ICR mice were used in each experiment. *P < 0.05.

To further investigate the subcellular localization of Rab27A, pancreas tissues from ICR mice were fractionated using percoll gradient centrifugation and ultracentrifugation. Samples from each fraction were analyzed by western blot. The subcellular compartments were labeled by respective specific markers: amylase for zymogen granules, Bip and calnexin for endoplasmic reticulum, voltage-dependent anion channel (VDAC) for mitochondria and LAMP1 for lysosomes. When compared with Rab27B and Rab3D, Rab27A was present in the zymogen granule membrane fraction, but in a much lower proportion to the amount of Rab27A in the initial homogenates (Fig 5A–5B). While Rab27A was present in most fractions similar to Rab27B, there was more Rab27A in the cytosol fraction than Rab27B (Fig 5A). Higher enrichment of Rab27A compared to Rab3D was detected in the mitochondrial and microsomal fractions (Fig 5B). These results further indicated that Rab27A was partially localized to zymogen granule membrane in the acinar cells, but was also localized to the less dense vesicular fractions, indicating its potential function in regulating secretion.

### Rab27A deficiency caused decreased amylase release in acinar cells

**Fig 6:**
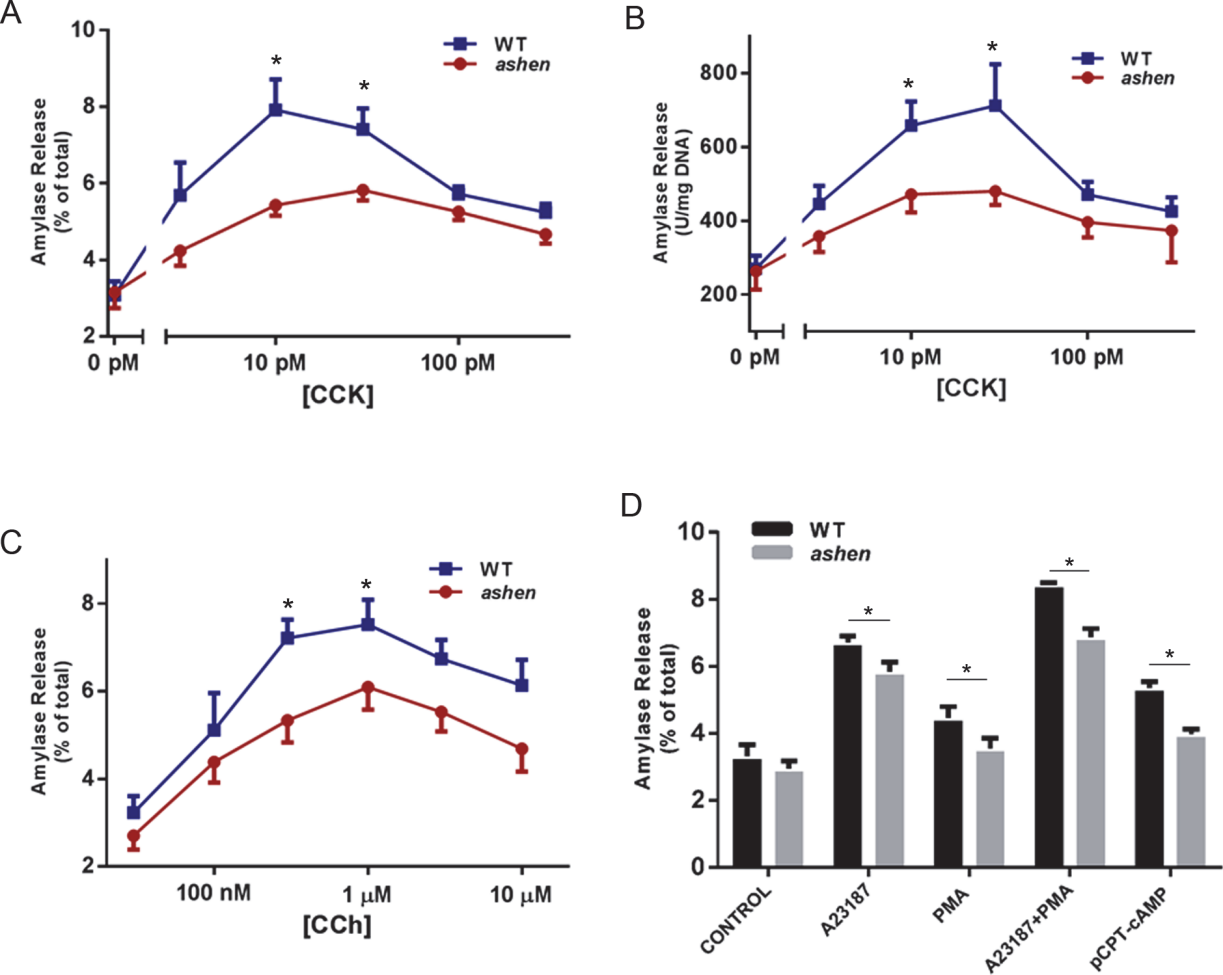
Rab27A deficiency caused decreased amylase release from isolated acini. (**A, B** and **C**) Freshly prepared acini were incubated with indicated concentrations of CCK (**A** and **B**) or carbachol (CCh, **C**) for 30 min. (**D**) Fresh acini were incubated with 2 μM A23187 alone, 500 nM PMA alone, a combination of 2 μM A23187 with 500 nM PMA or 100 μM 8-pCPT-2’-O-Me-cAMP (pCPT-cAMP) alone for 30 min. Amylase release results were expressed as percentage of total acinar amylase content (**A,C** and **D**) or released amylase units per mg DNA content (**B**). All results are mean ± SE from five independent experiments, respectively. One WT and one *ashen* mouse were used in each experiment. Each value was compared to the WT group at each concentration point of the indicated secretagogue. *P < 0.05.

To test the effects of Rab27A on amylase release, acini were isolated from WT or *ashen* mice and stimulated with a range of cholecystokinin (CCK) or carbachol (CCh) concentrations. Overall, *ashen* acini showed smaller fold increases in percent amylase release induced by CCK or CCh, especially at the peak concentrations of CCK or CCh (Fig 6A and 6C). This decrease is not caused by reduced amylase content in each acinar cell, as exhibited in Fig 6B, where amylase release is normalized to DNA content. When tested with other secretagogues, such as calcium ionophore A23187, Protein Kinase C activator phorbol-12-myristate-13-acetate (PMA) and cyclic AMP pathway activator 8-(4-Chlorophenylthio) adenosine 3’, 5’-cyclic monophosphate (pCPT-cAMP), *ashen* acini showed decreased secretion with all secretagogues, compared to WT. Although A23187 and PMA demonstrated synergistic effect in stimulating amylase release in both WT and *ashen* acini, *ashen* acini showed reduced secretion compared to WT (Fig 6D).

### Rab27A deficiency showed inhibitory effects on the lysosomal/endosomal secretory pathway but not ZG secretory pathway

**Fig 7:**
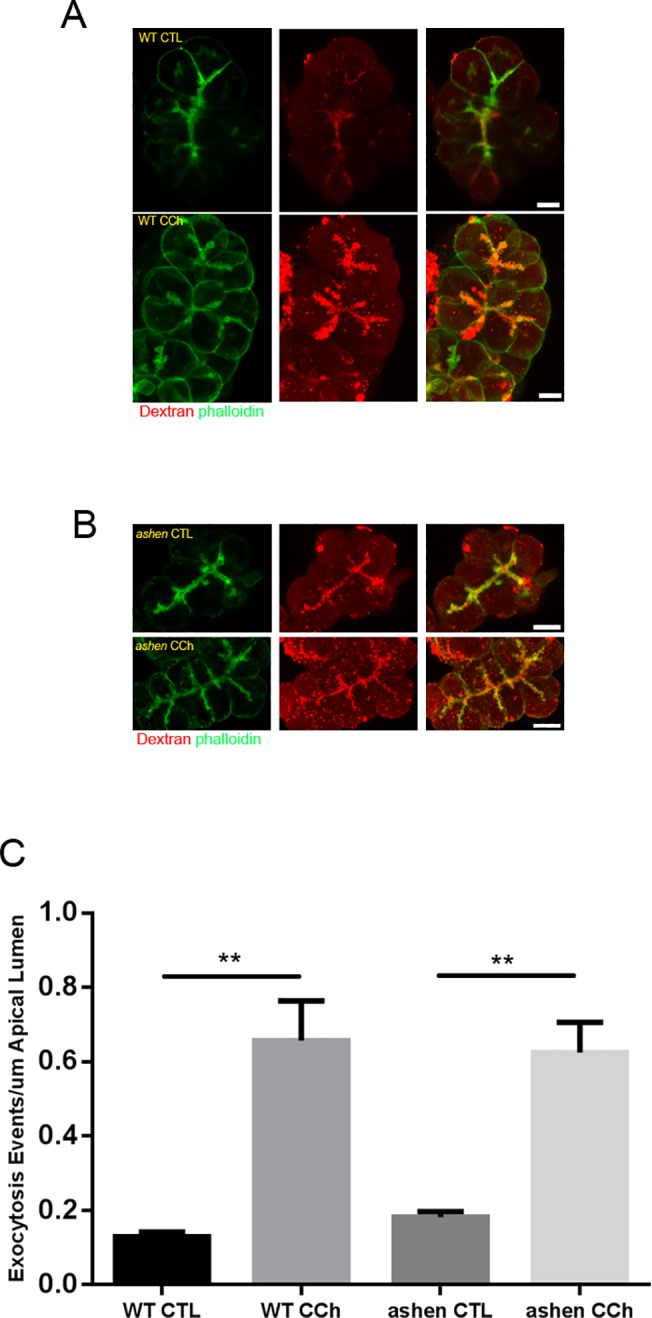
Visualization of zymogen granule secretory pathway by apical lumen marking with Dextran-Texas Red. (**A** and **B**) Freshly made acini were incubated with 1 mg/mL 3000 Da Dextran-Texas Red, stimulated with or without 3 μM carbachol (CCh), fixed and visualized as described in Material and Methods. Confocal microscopy images were obtained with a 60X objective lens. Representative maximum intensity projections of z-stacks images are shown. (**C**) Quantitative analysis on images obtained from 14 to 22 individual acini from three separate experiments. One WT and one *ashen* mouse were used in each experiment. Statistical results are mean ± SE. **P < 0.01. Scale bar = 10 μm.

**Fig 8:**
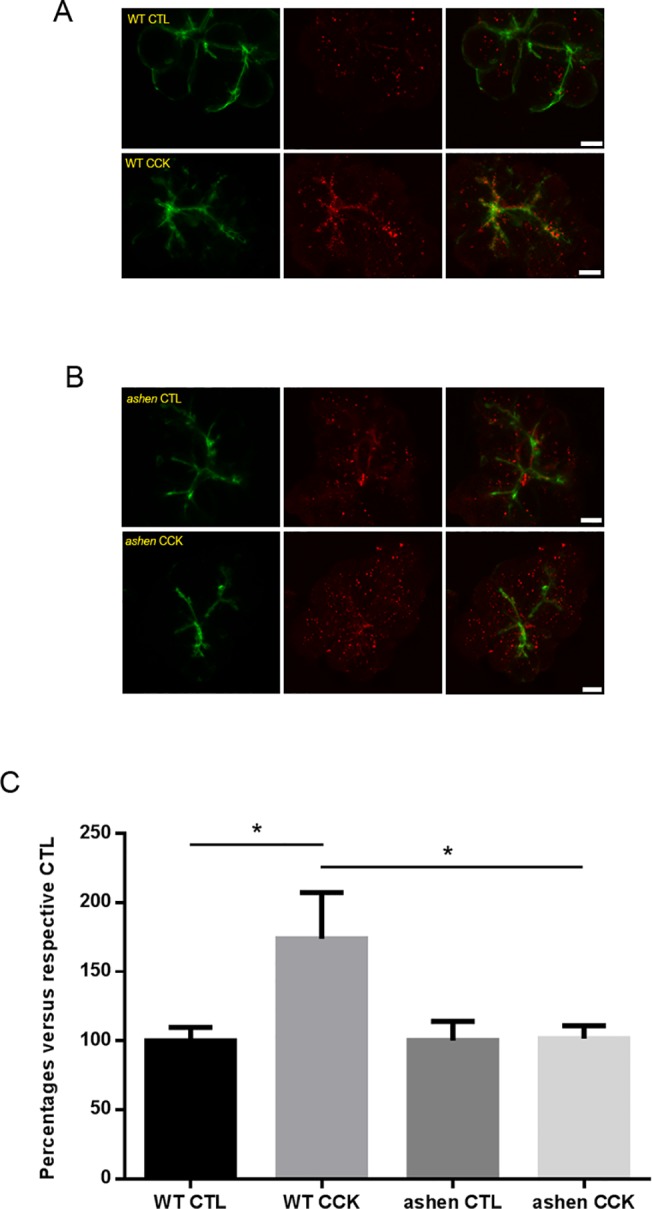
Visualization of minor-regulated secretory pathway by apical cell surface labeling with anti-LAMP1 staining. (**A** and **B**) Freshly made acini were stimulated with 30 pM CCK and processed as described in Material and Methods. Confocal images of LAMP1 staining (red) and actin (phalloidin, green) were obtained. Representative maximum intensity projections of z-stacks images are shown. (**C**) Quantitative analysis on the images obtained from 14 to 24 individual acini from three separate experiments. One WT and one *ashen* mouse were used in each experiment. Statistical results are mean ± SE. *P < 0.05. Scale bar = 10 μm.

To further investigate which secretory pathway Rab27A was involved with, secretion in isolated WT and *ashen* acini was compared by visualization of pathway specific fluorescent tracing. For the major zymogen granule secretory pathway, the exocytotic events were labeled by the incorporated luminal Dextran-Texas Red into the granule-plasma membrane fusion sites. In carbachol stimulated acini, multiple Texas Red positive dots were observed along the apical lumen and the majority of these dots were coated with phalloidin-labeled filamentous actin (Fig 7A–7B). Quantitative analysis showed that the numbers of exocytosis events at basal level or in CCh-stimulated *ashen* acini were similar to those in WT acini. Accordingly, the fold increases in secretion upon CCh stimulation were comparable between two groups (Fig 7C). Secretion through the lysosomal/endosomal mediated minor-regulated pathway was measured by evaluating the amount of fluorescence of externalized LAMP1 using an anti-LAMP1 antibody which reacts with an epitope externalized after fusion. LAMP1 staining showed specific localization along the apical lumen, which overlapped with apical phalloidin signals (Fig 8A–8B). CCK-treatment increased the intensity of apical LAMP1 in WT acini, compared with control, while in *ashen* acini no increase was observed (Fig 8C), indicating that LAMP1 externalization was impaired by Rab27A knockout. These results implied that Rab27A is involved in the minor-regulated secretory pathway, but not zymogen granule pathway. Although not studied in detail, LAMP1 labeling also showed some punctate staining in the cytosol, presumably due to CCK-induced endocytotic vesicle recycling (Fig 8A).

## Discussion

Both Rab27A and 27B are present in rodent pancreas, with Rab27A primarily in islets of Langerhans [10,29] and Rab27B in acinar cells [14]. Rab27B was originally identified in acinar cells, by mass spectrometry studies of proteins on the zymogen granule (ZG) membrane [14], which was further confirmed by western blot, immunofluorescence and quantitative proteomic analysis [12,13]. However, whether Rab27A is present in pancreatic acinar cells and its putative function was poorly understood. Rab27A and 27B are redundant to each other in function in some secretory cell types [30], while in other cells they control different steps of secretion [31,32]. When the sequences of Rab27A proteins from different mammals are compared, they show 95%-97% identity to the human Rab27A sequence, which indicates that Rab27A plays a conserved role in the secretory cells across the species [33]. In the present study, by utilizing *ashen* mice, which have a functional deletion of Rab27A, we demonstrated that Rab27A is present in pancreatic acinar cells and may regulate secretion at a different level than Rab27B.

Rab3D knockout pancreatic acini exhibit normal kinetics of amylase release [34], while *ashen* acinar cells showed decreased secretion. Given the data that Rab27A showed different sub-cellular localization than Rab27B and Rab3D, which are predominantly enriched on the ZG membrane, we hypothesize that Rab27A is involved in a different pathway. Castle et al [18] indicated that a minor-regulated pathway (MRP) has a secondary role in addition to mediating resting secretion, namely to provide the docking/fusion sites on the apical membrane for the ZG secretory pathway. Messenger et al showed that overexpression of D52 enhanced the endolysosomal secretory pathway in pancreatic acinar cells and increased secretion, as demonstrated by full dose response curve upon the CCK stimulation [17]. Their report, together with our findings, suggests that the decreased amylase release induced by different secretagogues in *ashen* acini may result from an impaired MRP. The hypothesized disruption to MRP in *ashen* acini was detected by anti-LAMP1 cell surface labeling, in which CCK-stimulated externalization of LAMP1 was probed by an antibody specific against the luminal epitope of LAMP1. In contrast, the dextran-Texas Red labeled ZG secretory pathway was not affected during the 5 min stimulation by CCh. These results indicate that Rab27A is mostly involved in MRP, and that Rab27A may play an overlapping roles with other small GTPases in regulating different steps of secretion in other cell types [11].

Imaging of exocytosis in pancreatic acinar cells by visualizing a luminal tracer, such as fluorescent dextran was developed by Nemoto et al in live acinar cells [26], and then applied to fixed acinar cells by Turvey et al [27]. In carrying out the Dextran-Texas Red labeling techniques in our lab, we performed the secretagogue-stimulation on glass slides and because CCK is more absorbent to glass than carbachol, we chose carbachol as a secretagogue. The assay had originally proved effective with the CCK-stimulation as well [26]. In contrast to zymogen granules, no successful purification of the vesicle populations that are exclusively secreted through MRP has been reported. However, lysosomal-associated membrane protein 1 (LAMP1), a single transmembrane protein is specifically present on the membrane of the endosomal/lysosomal-like compartment, with its N-terminal and the majority of the protein facing the lumen. Upon exocytosis, the luminal epitope of LAMP1 gets exposed to the extracellular domain after externalization and only this proportion of LAMP1 can be detected by anti-LAMP1 antibody under a non-permeabilization condition. Cell surface labeling assay by LAMP1 staining has been successfully adopted to demonstrate and quantify the secretion through MRP and constitutive-like pathways (CLP), because LAMP1 is not present in zymogen granule secretory pathway [17]. In Fig 8A and 8B, in addition to the staining along the apical lumen, the LAMP1 signals can be seen in the cytoplasm, being especially increased and intensified in CCK stimulated acini. We speculate this is due to the recycling of LAMP1 proteins that can be retrieved with bound antibodies from the plasma membrane during the CCK incubation. In addition to the roles in mediating exocytosis, GDP-Rab27A was shown to regulate endocytosis of insulin secretory membranes by promoting F-acting bundling [35–37]. In the present study, we did not observe obvious difference in the intensity or amount of punctate cytoplasmic LAMP1 staining, or of phalloidin staining of filamentous actin between *ashen* and WT acini.

In conclusion, we have shown that Rab27A is present in pancreatic acinar cells and involved in the regulation of secretion. In *ashen* mouse pancreas, the stimulated secretion was decreased, compared with WT. LAMP1-labelled minor-regulated pathway was down-regulated in *ashen* acini, while the zymogen granule secretory pathway marked by Dextran-Texas Red was not affected. All these data indicate that Rab27A is in mouse pancreatic acinar cells and regulates secretion through the minor-regulated pathway.

## Supporting Information

S1 Fig Scans of original western blot data used in the figures. (PDF)
