# Supplementary material for: Rab27A Is Present in Mouse Pancreatic Acinar Cells and Is Required for Digestive Enzyme Secretion
Source: PLoS One. 2015 May 7;10(5):e0125596. doi: 10.1371/journal.pone.0125596 (PMC4423933; doi:10.1371/journal.pone.0125596)

Original Image for Fig 1A anti-Rab27A

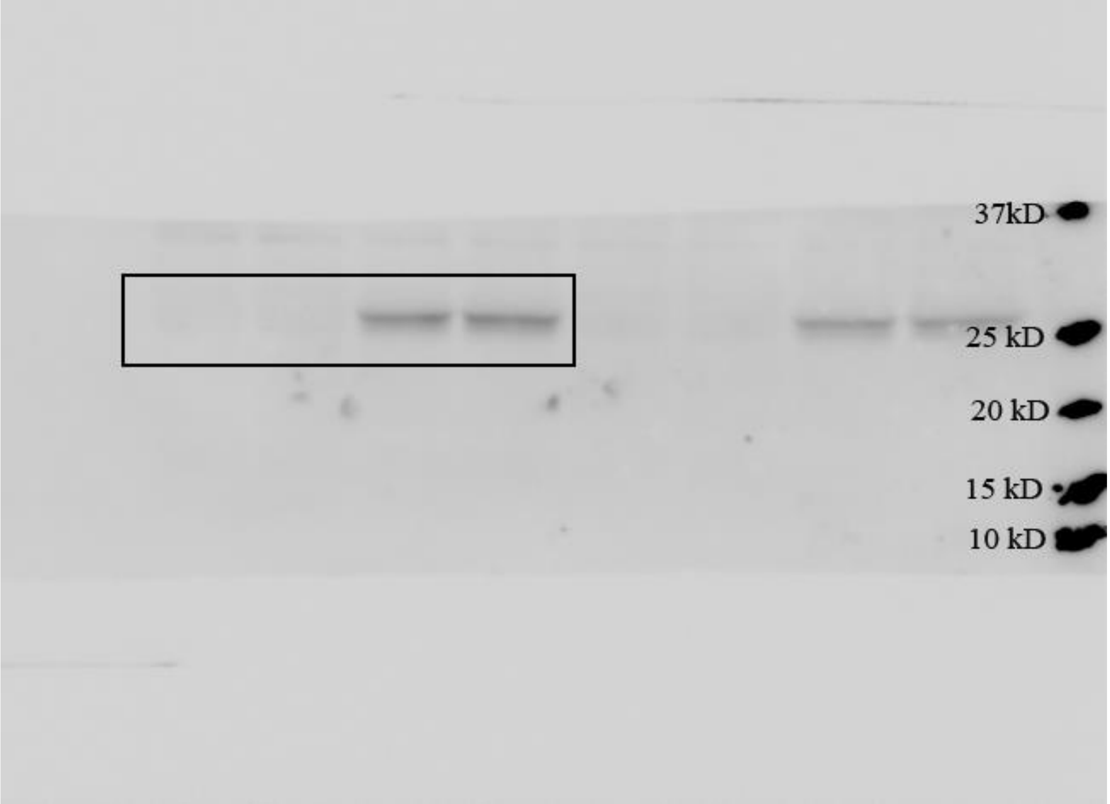

Original Image for Fig 1A anti-Rab27B

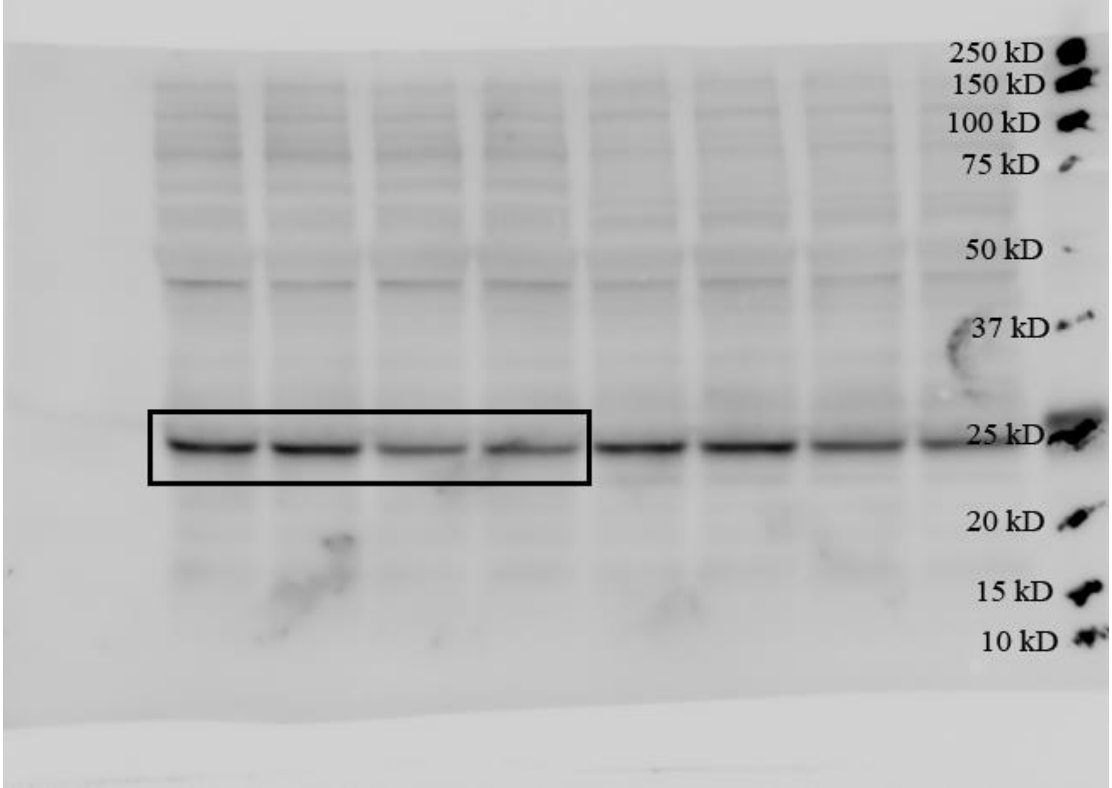

Original Image for Fig 1A anti-Rab3D

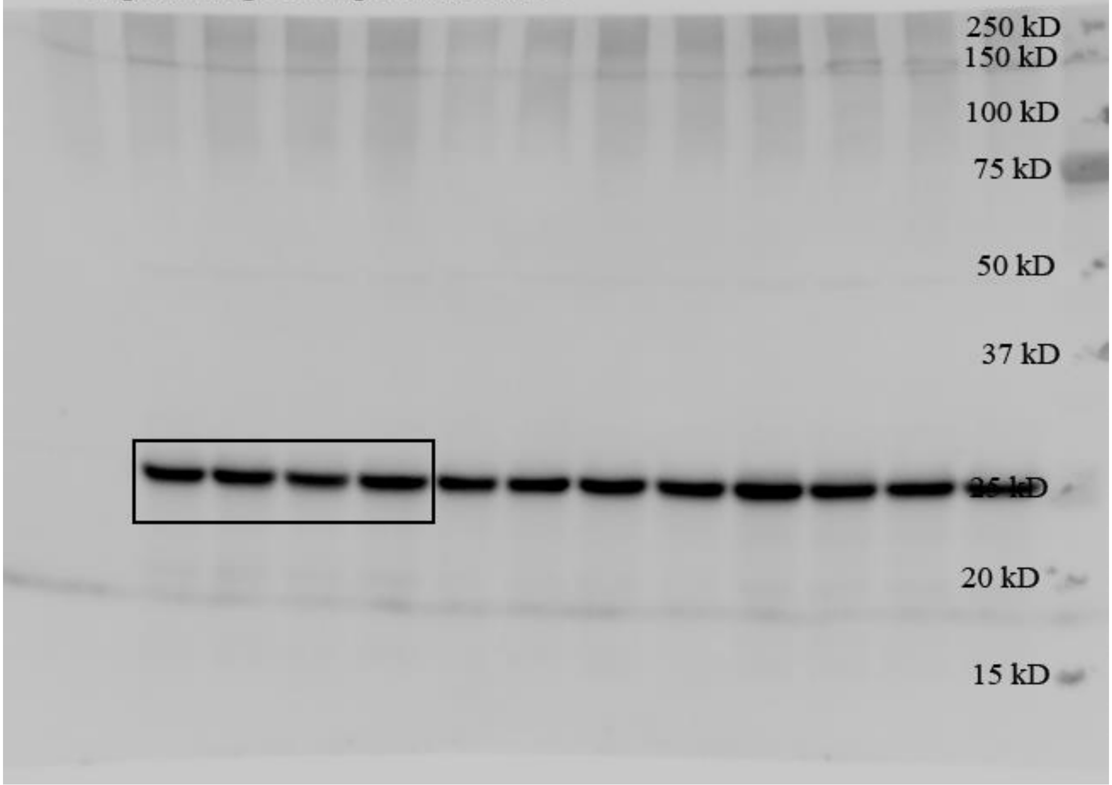

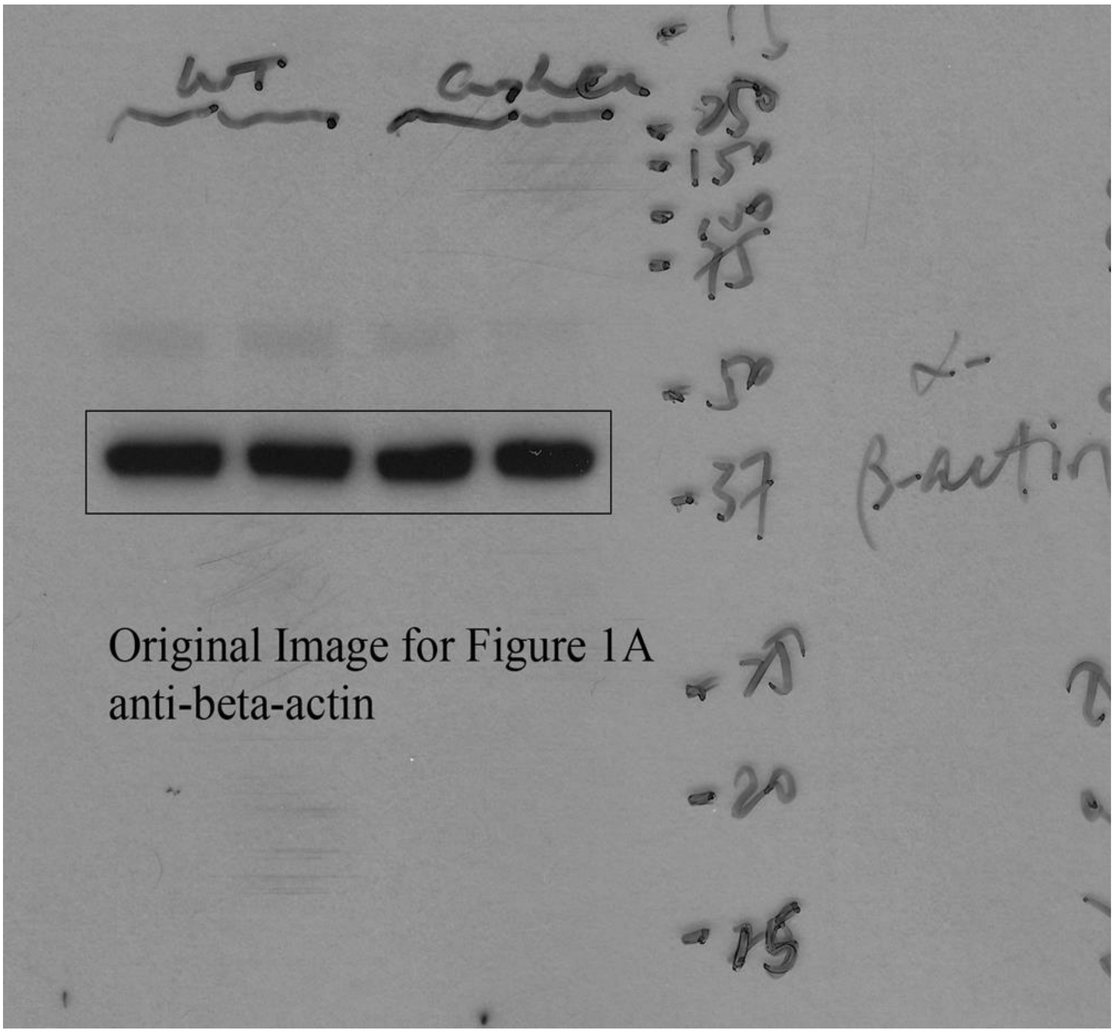

Original Image for Figure 1A
anti-beta-actin

Original Image for Fig 1C anti-Rab27B

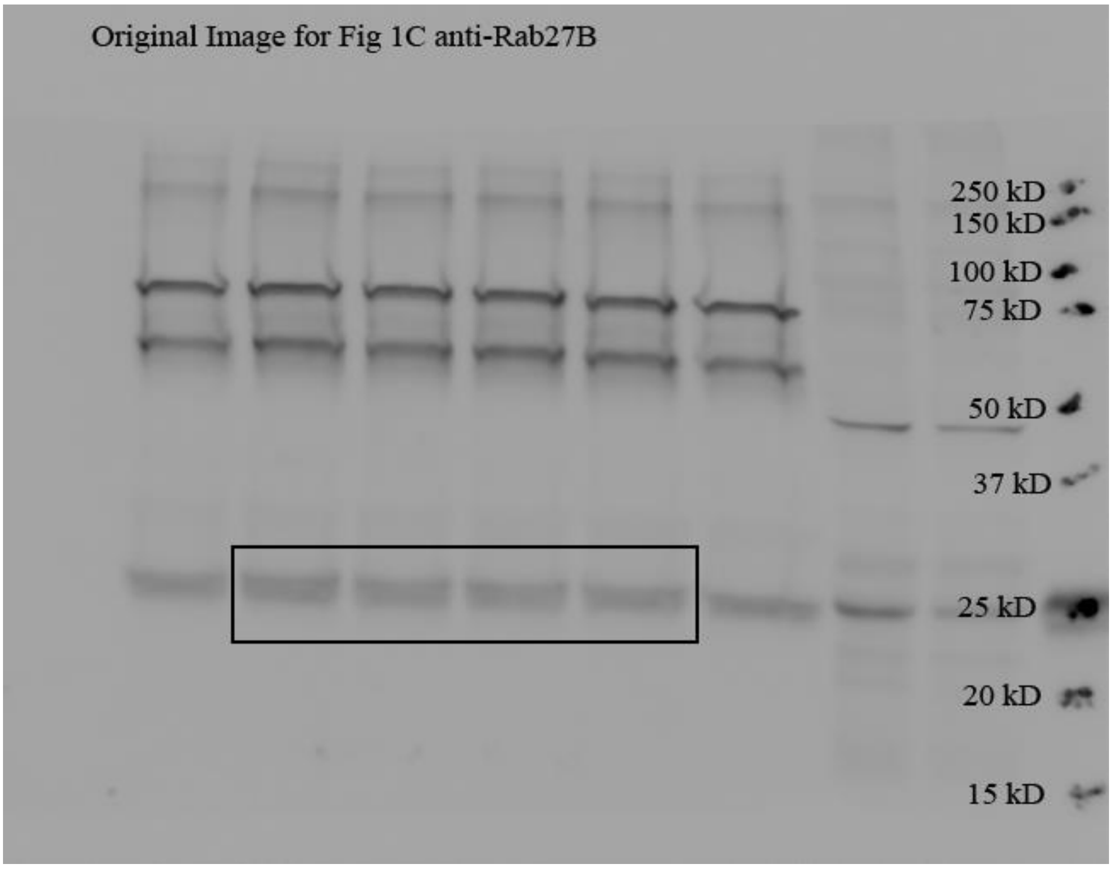

Original Image for Fig 1C anti-Rab3D

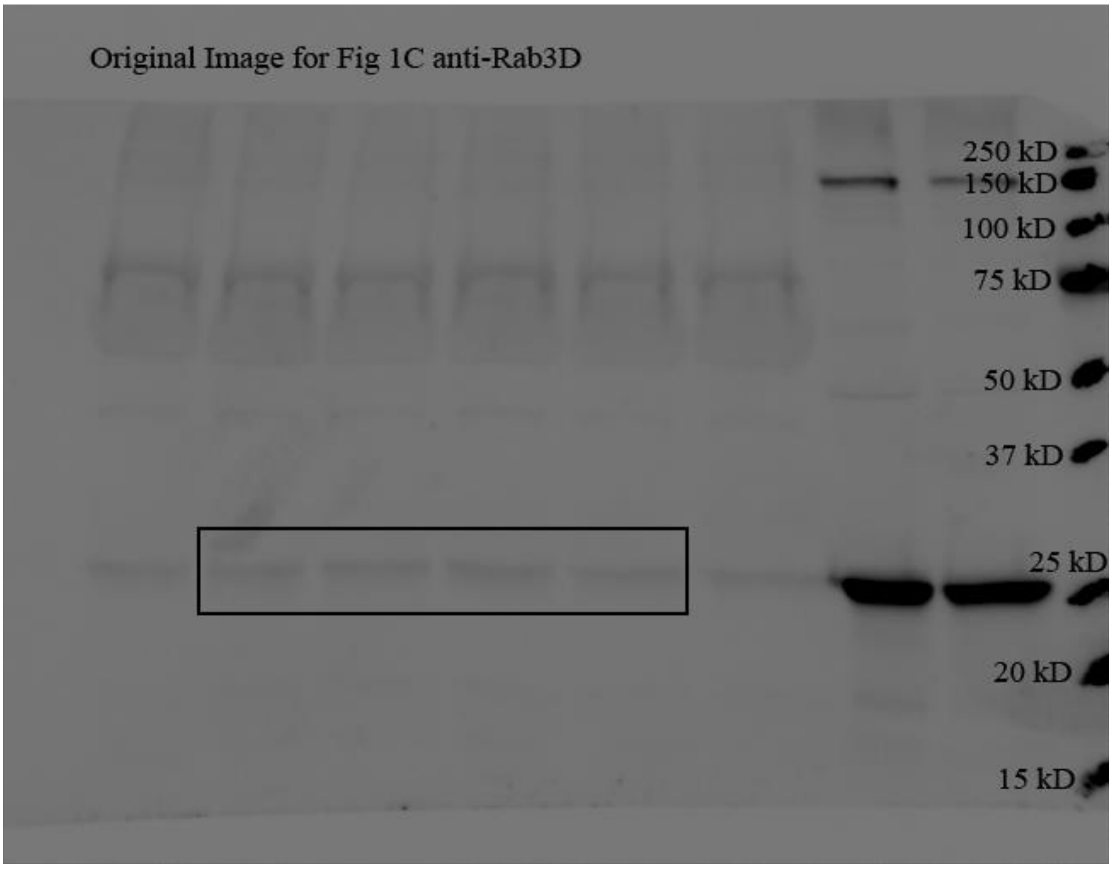

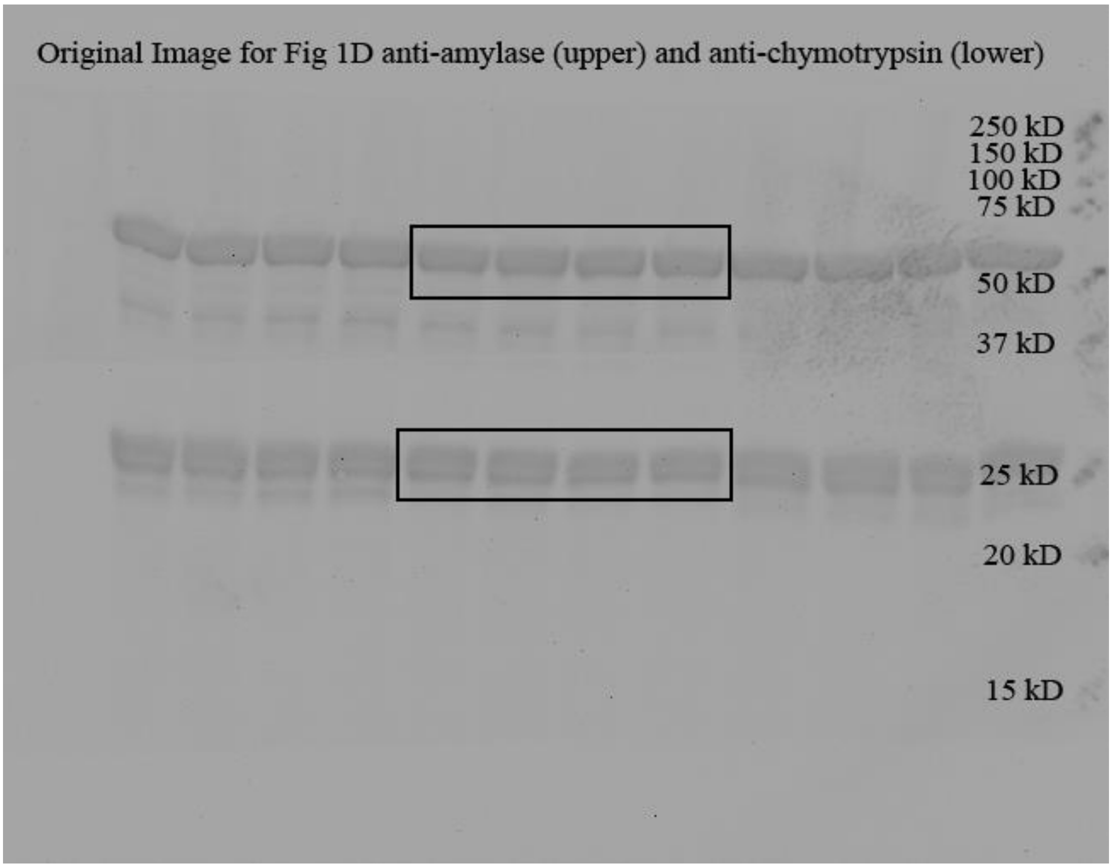
Original Image for Fig 1D anti-amylase (upper) and anti-chymotrypsin (lower)
250 kD
150 kD
100 kD
75 kD
50 kD
37 kD
25 kD
20 kD
15 kD

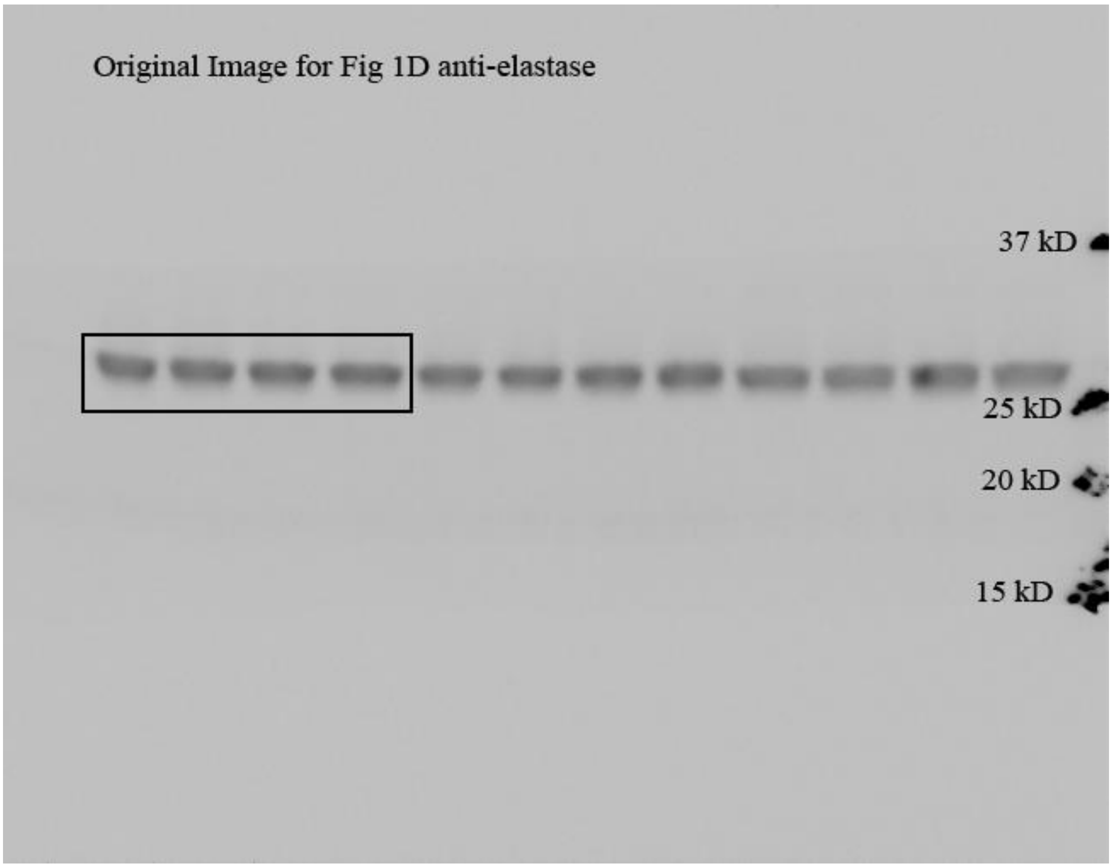
Original Image for Fig 1D anti-elastase
37 kD
25 kD
20 kD
15 kD

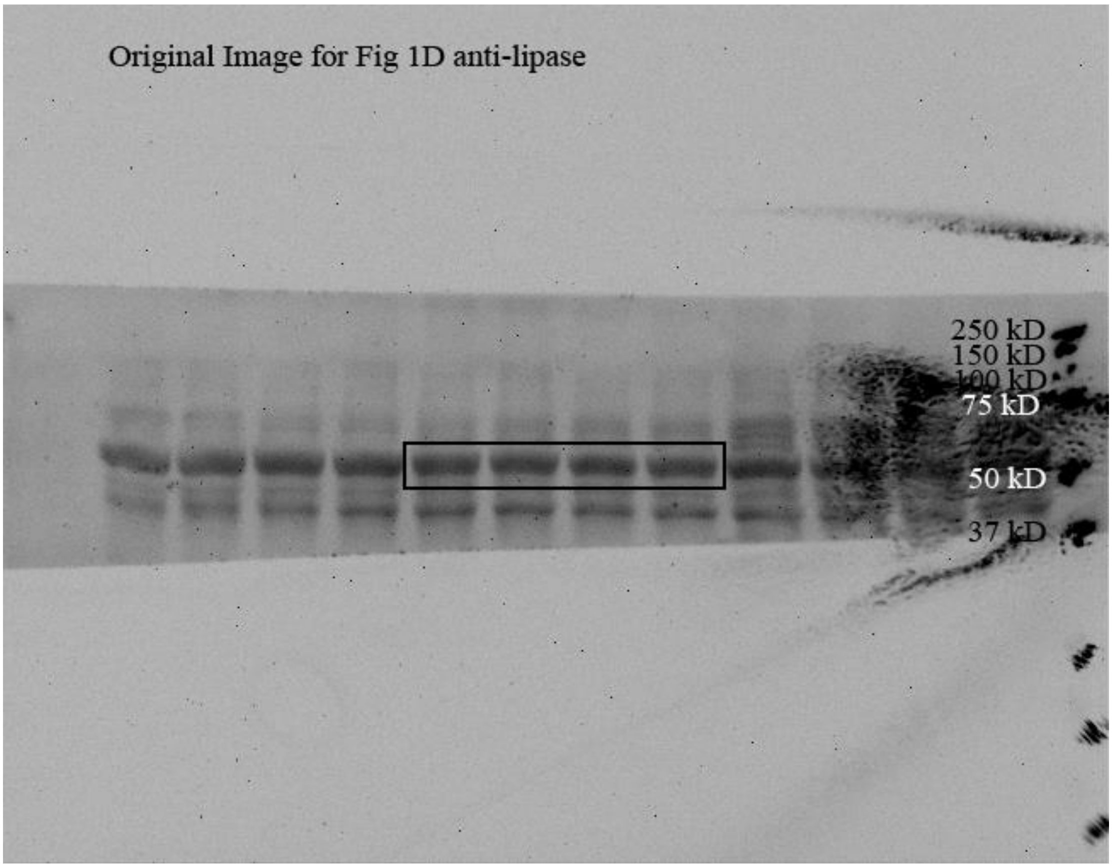
Original Image for Fig 1D anti-lipase
250 kD
150 kD
100 kD
75 kD
50 kD
37 kD

Original Image for Fig 1D anti-Rab6

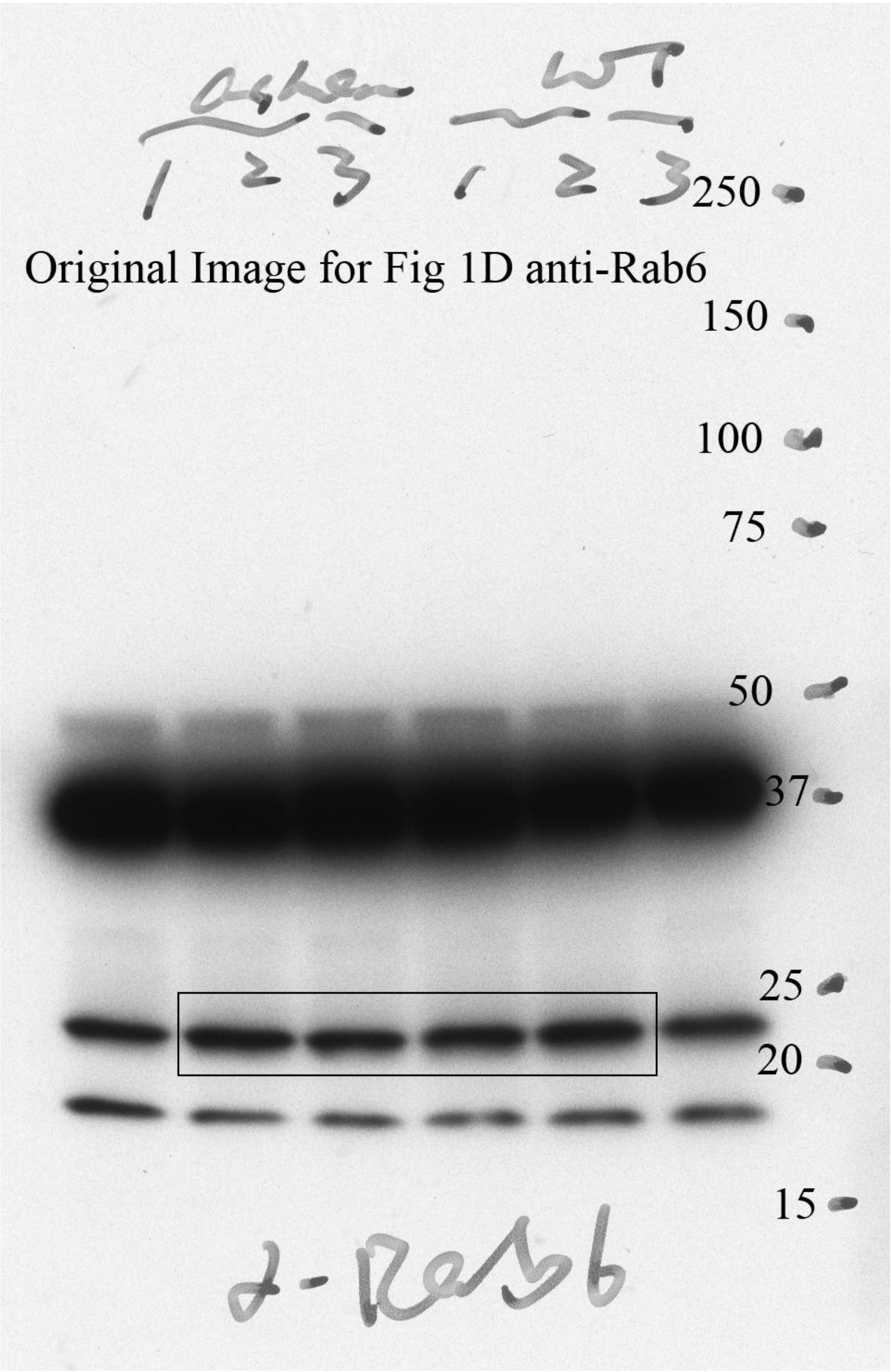

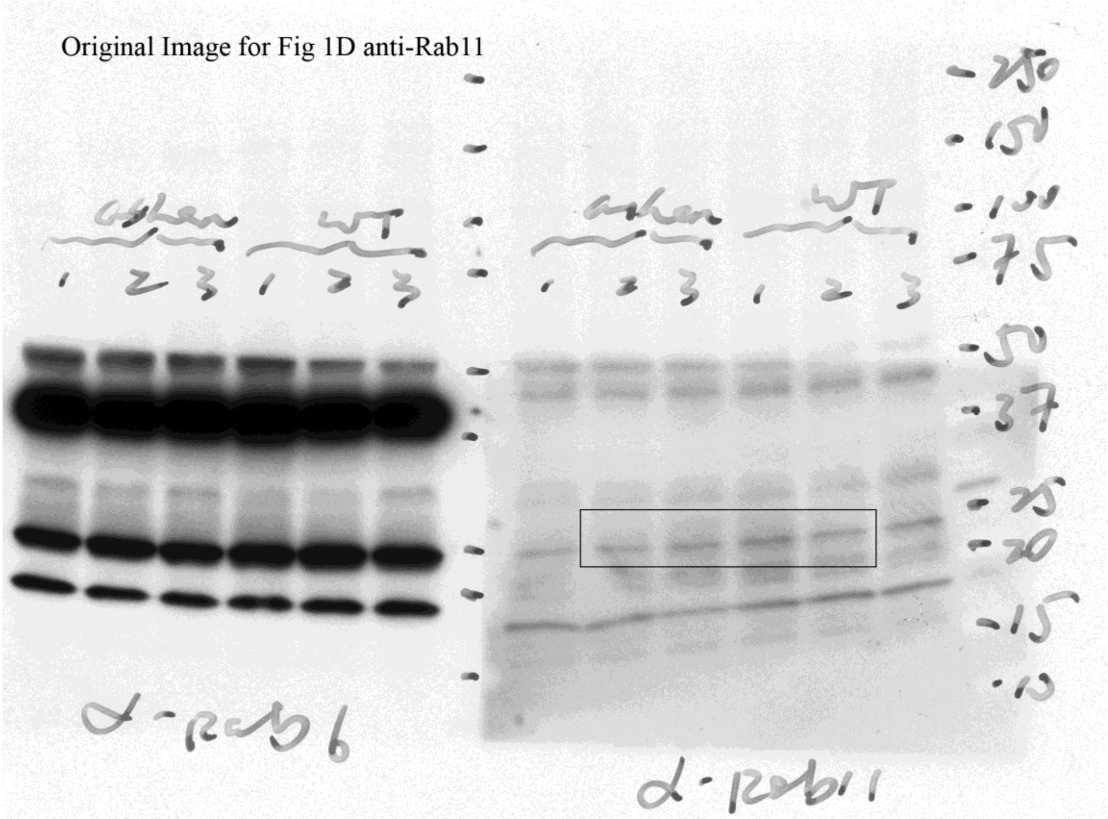
Original Image for Fig 1D anti-Rab11
ashen
WT
1 2 3 1 2 3
250
150
100
75
50
37
25
20
15
10
α-Rab6
α-Rab11

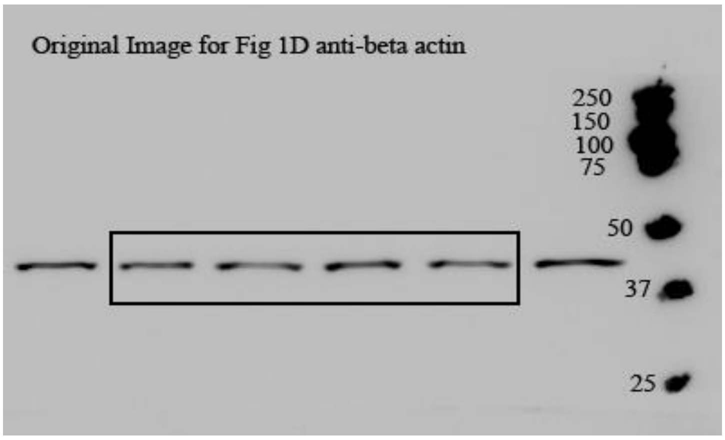
Original Image for Fig 1D anti-beta actin
250
150
100
75
50
37
25

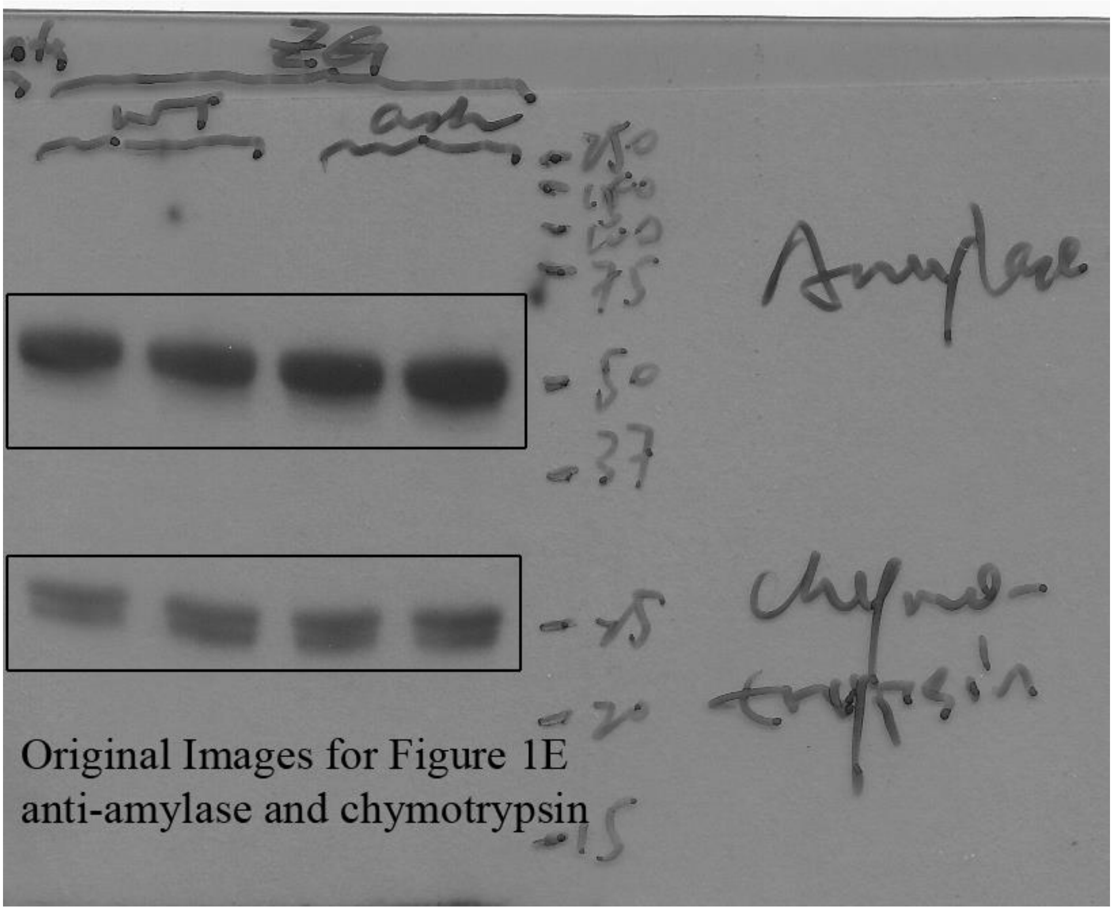

Original Images for Figure 1E
anti-amylase and chymotrypsin

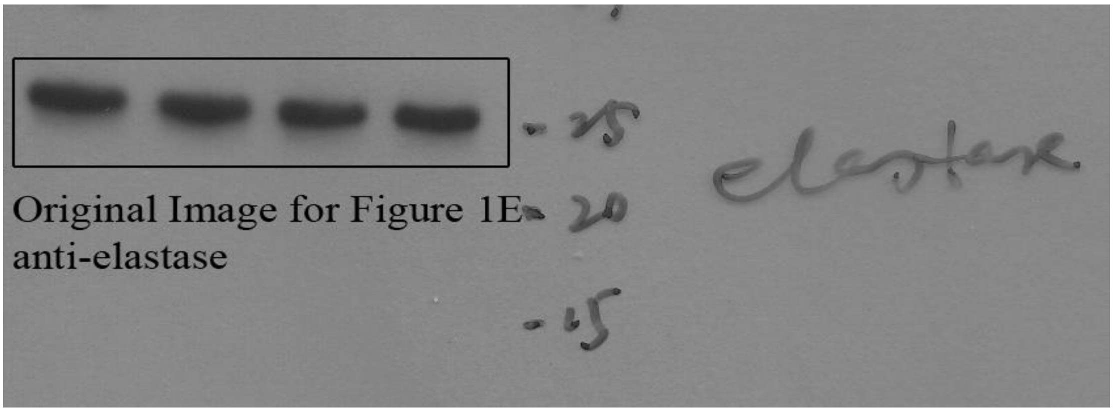

Original Image for Figure 1E
anti-elastase

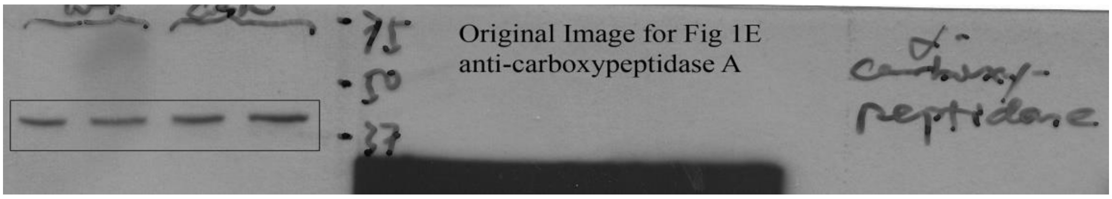

Original Image for Fig 1E
anti-carboxypeptidase A

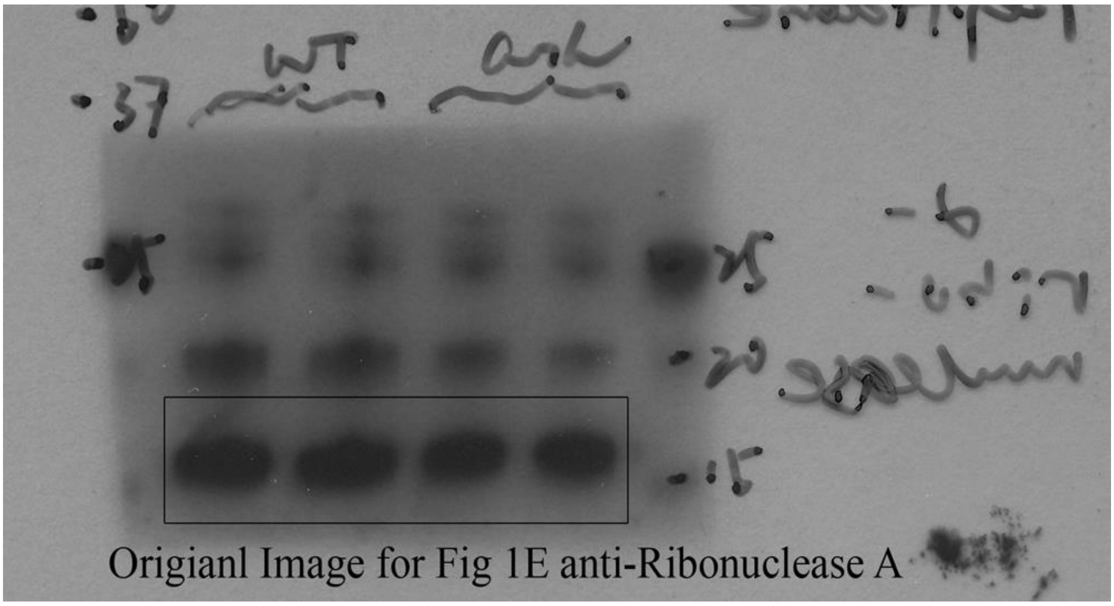

Origianl Image for Fig 1E anti-Ribonuclease A

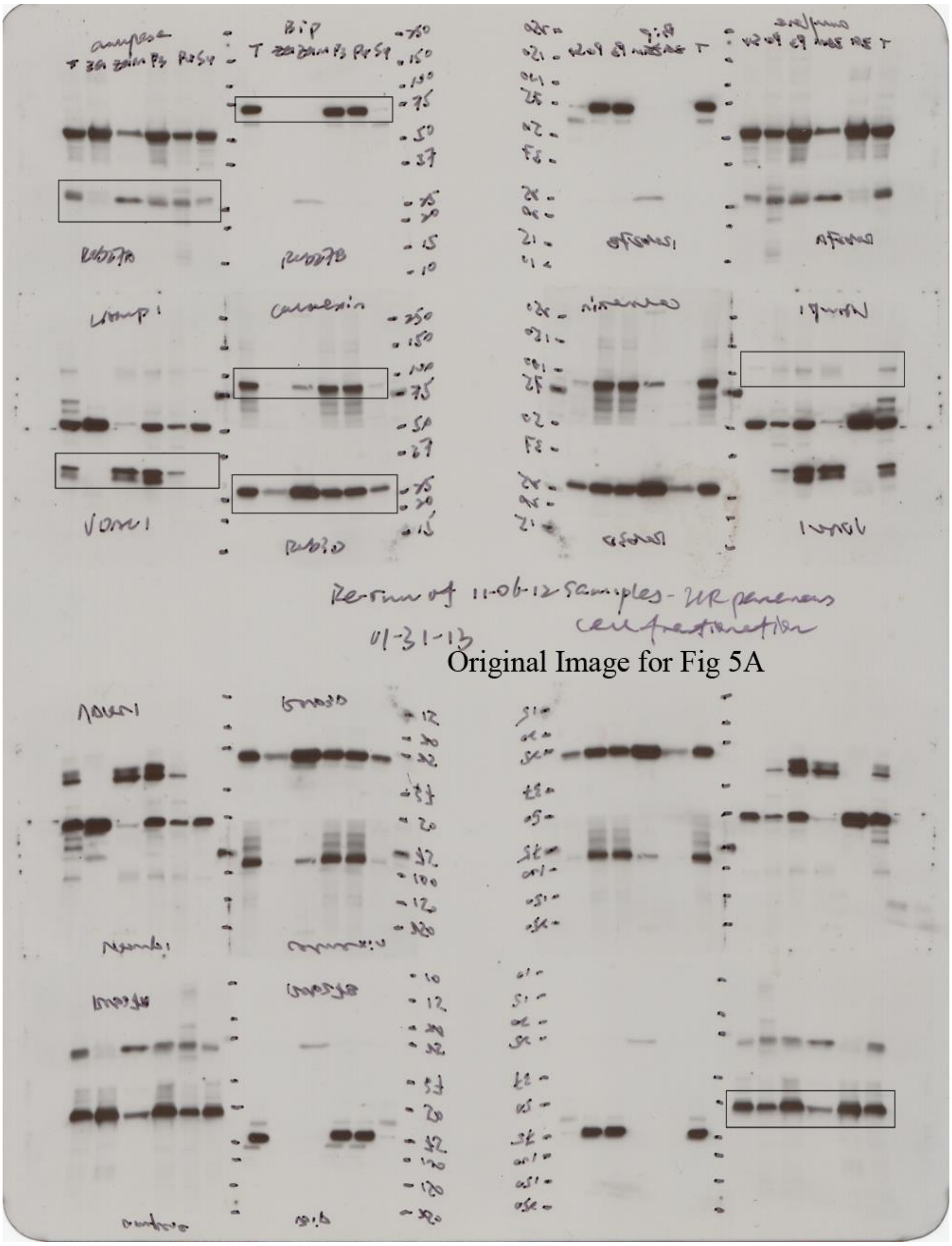

Original Image for Fig 5A

Original Image for Fig. 5A anti-Rab27B

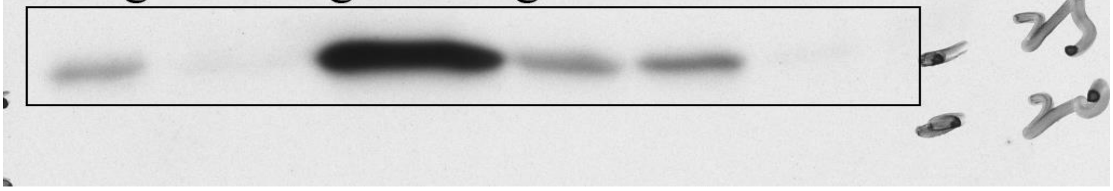

Supplement: S1 Fig — (PDF) [file pone.0125596.s001.pdf]
